# Water Behavior of Emulsions Stabilized by Modified Potato Starch

**DOI:** 10.3390/polym13132200

**Published:** 2021-07-01

**Authors:** Zuzanna Małyszek, Jacek Lewandowicz, Joanna Le Thanh-Blicharz, Katarzyna Walkowiak, Przemysław Łukasz Kowalczewski, Hanna Maria Baranowska

**Affiliations:** 1 Department of Food Concentrates and Starch Products, Prof. Waclaw Dabrowski Institute of Agriculture and Food Biotechnology—State Research Institute, 40 Starołęcka St., 61-361 Poznań, Poland; zuzanna.malyszek@ibprs.pl (Z.M.); joanna.lethanh-blicharz@ibprs.pl (J.L.T.-B.); 2 Department of Production Management and Logistics, Poznan University of Technology, 2 Jacka Rychlewskiego St., 60-965 Poznań, Poland; jacek.lewandowicz@put.poznan.pl; 3 Department of Physics and Biophysics, Faculty of Food Science and Nutrition, Poznań University of Life Sciences, 38/42 Wojska Polskiego St., 60-637 Poznań, Poland; katarzyna.walkowiak@up.poznan.pl; 4 Department of Food Technology of Plant Origin, Poznań University of Life Sciences, 31 Wojska Polskiego St., 60-624 Poznań, Poland; przemyslaw.kowalczewski@up.poznan.pl

**Keywords:** emulsion, nuclear magnetic resonance, LF NMR, relaxometry, modified starches, water dynamics

## Abstract

Starch is a widely known and used emulsion stabilizer. In order to improve its properties, various types of modifications are made that change its ability to emulsify and stabilize. This paper describes the analysis of the molecular dynamics of water using low-field nuclear magnetic resonance (LF NMR) in oil-in-water emulsions obtained with the use of physically or chemically modified potato starch. The analysis of changes in spin-spin and spin-lattice relaxation times depending on the temperature allowed the activation energy value of water molecules in the analyzed emulsions to be determined. It has been shown that the presence of starch influences the values of spin-lattice T_1_ and spin-spin T_2_ relaxation times, both in the water and the oil phase, and the observed changes largely depended on the type of starch modification. Both types of analyzed starches also differently influenced the energy of activation of rotational movements of water molecules. On the basis of the analyses carried out with the use of LF NMR, it can be concluded that physically modified starch acts not only as a stabilizer, but also as an emulsifier, while acetylated starch does not exhibit good emulsifying properties.

## 1. Introduction

Starch is one of the most abundant polysaccharides in nature, and is primary source of energy in our diet as well as a versatile, safe and cheap texture forming agent of food products. In this latter role, it is most often as used as modified starch preparation rather than in its native form. Starch can be modified by means of physical, chemical and/or biochemical methods. As a result, a wide variety of thickeners or gelling, bulking or water retention agents, as well as colloidal stabilizers, are available [1,2]. Hydrocolloids, including starches, are commonly used as stabilizers for oil-in-water emulsions. It is generally believed that hydrocolloids, due to their solubility in water and ability to form highly viscous solutions, immobilize oil droplets dispersed in a continuous water phase, and that way are active as emulsion stabilizers. Nevertheless, it is also noted that the suitability of the hydrocolloids for emulsion stabilization is determined, in some extent, by their surface activity [3,4].

The applicability of different types of modified starches is estimated most often using rheological methods as it is believed that the functionality of starch relates mainly to its ability to increase the viscosity of the system [5,6,7,8]. The effect of starch surface activity has been studied less frequently, mainly in relation to sodium starch octenylsuccinate, often called hydrophobic starch [9]. However, chemically modified starches show significant surface activity as the incorporation of substituents, which differ in hydrophilicity from the hydroxyl groups, promote adsorption of molecules at the interface [10]. Moreover, this activity can be increased inter alia by enzymatic hydrolysis [11,12,13,14]. Interestingly, it was also shown that the surface activity of starch was increased by physical modification [15]. This is probably due to the fact that physical modification can be accompanied by chemical changes [16]. Furthermore, the effect of molecular mass distribution on the functionality of modified starches in emulsions has been shown [17].

There is increasing interest in low field nuclear magnetic resonance (LF NMR), a method designed to study the dynamics of protons [18,19]. It makes it possible to study the molecular dynamics of water as well as phenomena occurring in fats [20,21], which opens new prospects of studying of complex food systems. It has been shown that LF NMR results correlate with the texture of food products [22,23,24,25,26]. Moreover, this technique is particularly precise regarding the phenomena occurring during starch gelatinization as well as correlating with the rheological properties of starch pastes [27]. Relaxation phenomena in starch pastes are much slower than in pure water or even in solution of low molecular mass substances, as water molecules in colloidal systems are entrapped by enormous polymeric molecules [28]. The process of nuclear relaxation in a spin system consists of transferring the energy accumulated in the system as a result of the action of an alternating magnetic field. Transverse relaxation is related to the transfer of energy between adjacent spins. The time it takes the system to return to thermodynamic equilibrium is called the spin-spin relaxation time and is denoted as *T*_2_. The transfer of energy to the environment requires a longer time called spin-lattice relaxation time and marked as *T*_1_. The addition of large molecules, the dynamics of which are much lower, slows down the rotation of water particles that bind at the sorption sites, thus, the relaxation time is shortened. It is known that in systems such as, for example, muscle tissue, water protons are found in different states depending on the mobility of the water fraction that can be defined as free, bound, bulk or entrapped [28]. The measured *T*_1_ and/or *T*_2_ relaxation times are then the weighted average of the relaxation rate

(1) $$\frac{1}{T_{1}}=p\frac{1}{T_{11}}+\left(1-p\right)\frac{1}{T_{12}}$$

(2) $$\frac{1}{T_{2}}=p\frac{1}{T_{21}}+\left(1-p\right)\frac{1}{T_{22}}$$

where: *p* is the fraction of protons associated with one type of water fraction, while *T*_11_, *T*_21_ as well as *T*_12_ and *T*_22_ are the relaxation times of individual proton fractions.

Relaxation times are macroscopic parameters. At the molecular level, the mobility of the studied molecules is demonstrated by a parameter called the mean correlation time *τ_c_*. It describes the time for a molecule to rotate by 1 radian. Bloembergen–Purcell–Pound (BPP) equations (Equations (3) and (4)) [29] contain the relationships between the spin-lattice *R*_1_ (=1/*T*_1_) and spin-spin *R*_2_ (= 1/*T*_2_) relaxation rates and the average correlation time *τ_c_*:

(3) $$R_{1}=\frac{1}{T_{1}}=\frac{6}{20}\frac{\mu_{0}^{2}}{16\pi^{2}}\frac{\gamma^{4}{\left(\frac{h}{2\pi }\right)}^{2}}{r_{0}^{6}}\left[\frac{\tau_{c}}{1+{\left(\omega \tau_{c}\right)}^{2}}+\frac{4\tau_{c}}{1+{\left(2\omega \tau_{c}\right)}^{2}}\right]$$

(4) $$R_{2}=\frac{1}{T_{2}}=\frac{3}{20}\frac{\mu_{0}^{2}}{16\pi^{2}}\frac{\gamma^{4}{\left(\frac{h}{2\pi }\right)}^{2}}{r_{0}^{6}}\left[3\tau_{c}+\frac{5\tau_{c}}{1+{\left(\omega \tau_{c}\right)}^{2}}+\frac{2\tau_{c}}{1+{\left(2\omega \tau_{c}\right)}^{2}}\right]$$

where: *μ*_0_ is the permittivity of free space, *γ* is the magnetogyric ratio, *h* is the Planck constant, *r*_0_ is the distance of the interacting nuclei, and *ω* is the resonance frequency (*ω* = 2π*f*, *f* is the Larmor frequency, the spectrometer frequency).

The above equations are valid for a system characterized by one mean correlation time *τ_c_*, when molecules or functional groups perform rotational movements with a constant frequency, conditioned by their interaction with the environment at a specific temperature. If the rotational movements are thermally activated, it means that the molecules or ions must cross the potential barrier of the height Δ*E_a_*, which separates the adjacent minima of potential energy. The temperature dependence of the correlation time is described by the Arrhenius equation and has the form:

(5) $$\tau_{c}=\tau_{0}exp\left(\frac{\Delta E_{a}}{RT}\right)$$

where: Δ*E_a_* is the energy barrier to the rotation of molecules, *R* is the gas constant, and *T* is the temperature on an absolute scale.

LF NMR shows particular advantages in studies of emulsions due to the strong correlation between their stability and relaxation phenomena occurring in both the oil and the water phase. Due to formation of an emulsion, which is a two-phase system, two components of both relaxation times are observed; the long components (*T*_12_ and *T*_22_) that reflect proton the relaxation processes in the water phase and the short components (*T*_11_ and *T*_21_) related to the oil phase of the emulsions [30,31,32,33]. The presence of two components of relaxation times means that the protons in the tested system are in two relaxing phases with different times. The long components of *T*_12_ and *T*_22_ have values similar to those characteristic of starch pastes of a similar concentration of starch in water and recorded at a frequency of 15 MHz [30,31,32,33]. Additionally, long components of spin-lattice relaxation times take larger values than the corresponding components of spin-spin relaxation times, while short components *T*_11_ and *T*_21_ have similar values and additionally similar to those characterizing fats measured at low Larmor frequency [34,35,36]. Therefore, long components of the relaxation times were ascribed to the water phase of the emulsion, and the relaxation of the protons of the oil phase describe the short components of both relaxation times. Detailed changes in their values enable hypotheses to be made about the physicochemical phenomena occurring in emulsions.

There is increasing skepticism among consumers regarding the use of food additives, including chemically modified starches, in spite of the fact that they are safe and could be used according to *Quantum satis* principle [37,38]. Customers avoid buying foods whose producers declare to use substances that have been assigned with an E number. In response to this skepticism, the clean label movement has emerged and manufacturers have begun to look for a natural alternative to the currently used additives, including native and physically modified starches [8].

Considering the popularity of the clean label trend and the usefulness of the LF NMR technique in the analysis of emulsion systems, studies on molecular properties of emulsions stabilized with acetylated or physically modified potato starch were carried out. It was stated that both types of starches are able to stabilize emulsions; however, in contrast to acetylated starch, physically modified starch acts in studied emulsions not only as a stabilizer but also as an emulsifier or fat replacer.

## 2. Materials and Methods

### 2.1. Emulsions

Beef or porcine fats (Animex Foods sp. z o.o., Morliny, Poland) were used to form the oil phase of the studied oil-in-water type emulsions. Chemically modified starch E1420 (WPPZ S.A., Luboń, Poland) or physically modified starch LU 1432 (WPPZ S.A., Luboń, Poland) produced according to Polish Patent No. 207800 were applied for the formation of water phases. As the studied fats were solid at the room temperature, both the oil and water phases (starch suspensions) were heated to the temperature of 40 °C before mixing. Then, the resulting dispersion was heated to the temperature of 90 °C under constant stirring. The emulsions were formed along with the starch pasting process. The heat treatment at the temperature of 90 °C under constant stirring, using a mechanical stirrer MS-H-Pro (ChemLab, Kielce, Poland), continued for one hour. The ratio of oil to water was set to 1/4 in all emulsions, and the concentration of the starch in water phase was set to 0.12 g, 0.17 g and 0.25 g (*w*/*w*). Freshly prepared samples were put in the NMR tubes, closed by Parafilm^®^ and placed in the NMR spectrometer.

### 2.2. Fatty Acid Profile

The fatty acid composition was determined by the gas chromatography technique according to the ISO 17059 standard [39]. The chromatographic separation was performed using a Hewlett-Packard 5890 SII gas chromatograph equipped with a Supelcowax 10 capillary column (30 m × 0.25 mm × 0.25 μm) and a flame ionization detector (FID). The injector temperature was 240 °C, and the detector temperature was 240 °C, with the programmed furnace temperature: maintained at 60 °C for 1 min, then an increase of 12 °C/min to 220 °C, maintained for 25 min. Fatty acids were identified from the retention times of the standards.

### 2.3. NMR Measurements

Measurements of relaxation times (spin-lattice T_1_ and spin-spin T_2_) were performed using pulse NMR spectrometer PS15T (Ellab, Poznań, Poland) operating at 15 MHz, equipped in system of temperature control.

The spin-lattice relaxation times T_1_ were measured using inversion-recovery sequence 180−τ−90 [40]. Spin-lattice relaxation times were calculated with the assistance of the CracSpin program [41], allowing a multiexponential magnetisation recovery to be calculated. The spin-spin relaxation times T_2_ were measured using a CPMG impulse sequence (90−τ−180_n_) [42,43]. The parameters of pulse sequence were adjusted to starch concentration and temperature. Five accumulation signals of spin echo sequences were applied. Calculations of relaxation times were made by adjusting the recorded delays of spin echo amplitudes to the formula, which took into consideration the multiexponential delay. Measurements were carried out at temperatures ranging from 90 °C to 0 °C (±0.5 °C).

## 3. Results and Discussion

Table 1 shows the fatty acid profile of the fats used in the research. They are typical for the analyzed fats and are consistent with the literature data [44,45]. Two saturated acids (palmitic and stearic) constitute a total of 47.9% of the composition of beef fat and 41.6% of the composition of pork fat. Unsaturated oleic and linoleic acids account for another 43.0% and 49.0% of the composition of these fats, respectively. Thus, the effect of the type of fat on the water dynamics in water phase of emulsions is dominated by these four fatty acids. It is worth noting that in pork fat, both saturated fatty acids (C16:0 and C18:0) have the same content as the C18:1 unsaturated acid. Beef fat has about 10% more saturated fatty acids (C16:0 and C18:0) than pork fat, which makes it a harder fat [46].

**Figure 1:**
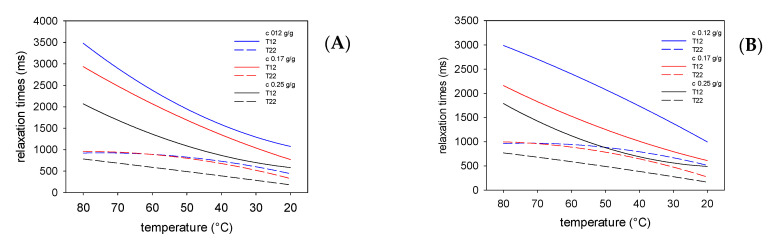
Changes in the spin-lattice and spin-spin relaxation times in the water phase, during cooling of the emulsions stabilized with E 1420 starch containing beef (**A**) and pork (**B**) fat.

**Figure 2:**
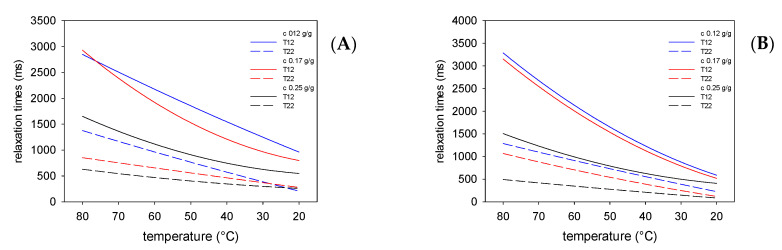
Changes in the spin-lattice and spin-spin relaxation times in the water phase, during cooling of the emulsions stabilized with LU1432 starch containing beef (**A**) and pork (**B**) fat.

**Figure 3:**
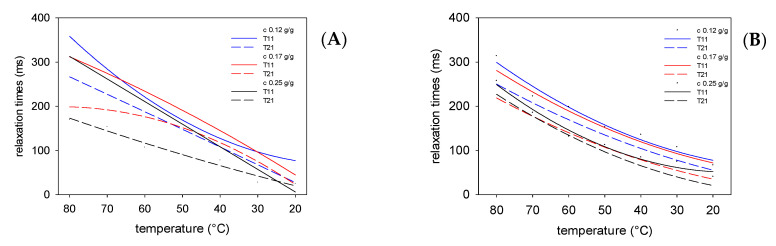
Changes in the spin-lattice and spin-spin relaxation times in the oil phase, during cooling of the emulsions stabilized with E1420 starch containing beef (**A**) and pork (**B**) fat.

**Figure 4:**
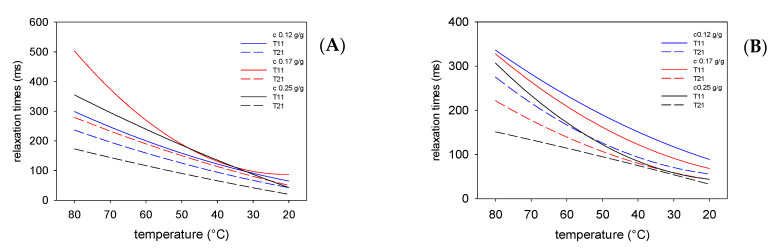
Changes in the spin-lattice and spin-spin relaxation times in the oil phase during cooling of the emulsions stabilized with LU1432 starch containing beef (**A**) and pork (**B**) fat.

The preparation of the emulsion requires the use of an emulsifier a substance capable of reducing the interfacial tension at the oil–water interface. It is believed that starch as a polysaccharide that does not reveal surface activity acts only as a stabilizer and extends the durability of this two-phase dispersed system [10]. Previous studies on the application of native potato starch have proven that it is possible to prepare stable emulsions without an surface active agent, however high the concentrations of starch that are required; at least 0.17 g/g in the water phase [31,32]. Acetylated starch E 1420 analyzed in this study shows the highest surface activity (except sodium starch octenyl succinate E 1451) among popular food grade modified starches [10]. Surface activity of the tested physically modified starch was not analyzed; however, it is known that some physically modified starches are useful in forming an emulsion [15]. Despite the above, it should be mentioned that, in certain conditions, modification may lead to deterioration of starch emulsion capacity [47]. The NMR experiment presented in Figure 1, Figure 2, Figure 3 and Figure 4 proved that the presence of starches in the water phase of the emulsion affected all measured relaxation times. The kind and the extent of these changes depended on the type and concentration of starch. The presence of starch macromolecules in the water phase (Figure 1 and Figure 2) resulted in a decrease in the relaxation times compared to those reported previously [27,31]. Moreover, the increase in concentration of starch results in a decrease in relaxation times that is an effect of the entrapping of water molecules by the colloidal lattice [28]. However, the effects of chemically (E 1420) and physically (LU1432) modified starches were different. Physically modified starch at the highest concentration caused a significantly higher decrease in spin lattice relaxation times than at both lower concentrations studied. Moreover, the decrease in spin lattice relaxation times in water phases at the highest starch concentration was greater for physically modified preparation compared to that modified chemically. It should be also noted that the type of oil phase also affects the relaxation times, but this effect is not as significant as that of starch. Similar conclusions can be drawn from rheological data published by Jo et al. [48], where pregelatinized OSA preparation at the highest concentration affected the consistency index of emulsions to a larger extent. Furthermore, the effect of oil concentration was less significant when compared to starch.

The type and the concentration of starch also affected the relaxation times in the oil phase of the emulsions, although this effect was smaller than in the water phase. This observation suggests that starch, although not dissolved in the oil phase, revealed an entrapping effect on fat molecules. This may be related to the higher viscosity of the emulsion than that of the oil phase, caused by the presence of the viscous water phase. The course of the changes in the values of relaxation times affected by type of fat used was more evident in the oil phase than in the water phase. This is especially evident for emulsions stabilized with chemically modified starch.

For a detailed analysis of the molecular dynamics of proton-containing molecules in starch-containing emulsions, the protons in the oil phase and water phase were analyzed separately. Using analytical solutions of BPP equations (Equations (3) and (4)) [29], taking into account mean correlation times, temperature changes in this parameter value for individual emulsions were determined. Then, using Equation 5, the energy barrier values of the rotational movements of molecules containing protons were determined. Due to the fact that protons in the analyzed emulsions are in two independent phases (oil and water), the values of the energy barrier for these two phases were determined separately, and the results of the calculations are presented in Table 2.

It was shown that, when using chemically modified starch (E 1420), the molecular dynamics of the oil phase were characterized by an energy barrier approximately twice as high as the oil phase. Similar results were obtained for emulsions containing animal fat and native potato starch [31]. The use of physically modified starch (LU1432) caused a significant effect of this polymer on the molecular properties of protons in the emulsion. The oil and water phase were characterized by similar, high values of the energy barrier of the rotational movements of molecules. The oil phase of emulsions obtained from both types of fats were characterized by similar values of the activation energy of rotation movements. It was lower for pork fat than for beef fat, due to different fatty acid content. The more saturated fatty acids are, the more ordered the structure of the fat [49,50]. This leads to the inhibition of the rotational movements. For both analyzed starches, similar values of ∆*E_a_* of the oil phase were recorded; however, both fats used in the research are characterized by a high content of C16:0 and C18:0 fatty acids. It is known, however, that in the presence of water, chains of fatty acids in emulsions create specific lamellar structures, and the use of E1420 starch eliminates the differences between the fatty acid composition of pork and beef. It should also be noted that *E_a_* correlates well with the temperature-dependent rheological proprieties of the starch pastes including those derived from both rheometers and viscorpaghs [51]. Moreover, it was shown by Zhang et al. that the chemical modification of starch with OSA led to an increase in emulsion stability, along with RVA parameters such as breakdown and setback thermal stability (that partially indicate the thermal stability of starch paste) [52].

As shown in Table 2, the oil phases of emulsions obtained using both fats were characterized by similar values of the activation energy of rotation movements; however, slightly lower values were recorded for pork than for beef fat. This could be due to the different fatty acid content. The more saturated palmitic and stearic acids, the more orderly the structure of the fat [49,50]. This leads to the inhibition of the rotational movements. A much stronger differences in the effect of the presence of different starches was observed in the water phases. When using chemically modified starch (E1420), the molecular dynamics of the oil phase were characterized by an energy barrier approximately twice as high as that of the water phase. Similar results were obtained for emulsions containing animal and native potato starch [31]. When using physically modified starch (LU1432), the oil phases obtained with both types of fats were characterized by similar values for the activation energy of rotation movements. The observed effects can be explained by the different molecular structure of the studied starches. In spite of some surface activity of acetylated starch, its molecular structure, in terms of molecular mass distribution and degree of branching, is almost identical to that of unmodified starch. Moreover, the radius of gyration and hydrodynamic radius are also not affected by acetylation [53]. As a consequence, acetylated starch should be considered simply as a stabilizer of the emulsions. On the other hand, it is known that physical modification affects molecular mass distribution, degree of branching, radius of gyration, hydrodynamic radius and even surface activity of starch [15,54,55]. Moreover, taking into consideration the strong effect of physically modified starch on spin-lattice relaxation time, especially in high concentrations (Figure 2), as well as the fact that there was almost no difference between relaxation times in both oil and water phases, one can conclude that physically modified starch is not only an emulsion stabilizer, but also an emulsifier or fat replacer.

## 4. Conclusions

The presence of starch in water phase of the emulsion affects spin-lattice and spin-spin relaxation times in both the water and the oil phases. The kind and the extent of these changes depends on the concentration and type of starch. The most noticeable difference was observed for spin lattice relaxation times in water phase, which was higher for physically modified starch. Physically and chemically modified starches affected the activation energy of rotational movements of molecules in different ways. LF NMR studies indicated that acetylated starch acted as emulsion stabilizer. In contrast, physically modified starch can be recognized as not only a stabilizer, but also as an emulsifier or fat replacer in emulsion studied. Relaxation phenomena are also partially affected by the fatty acids composition in studied fats.

## Figures and Tables

**Table 1:** Fatty acid profiles expressed in [%].

Fatty acid	Beef Fat	Pork Fat
C 10:0 capric acid	0.043 ± 0.001	0.059 ± 0.001
C 12:0 lauric acid	0.058 ± 0.001	0.073 ± 0.001
C 13:0 tridecanoic acid	0.012 ± 0.001	N/D
C 14:0 myristic acid	2.622 ± 0.020	1.365 ± 0.009
C 14:1 oleomyristic acid	0.423 ± 0.003	0.068 ± 0.001
C 15:0 pentadecanoic acid	0.499 ± 0.001	0.117 ± 0.001
C 16:0 palmitic acid	26.493 ± 1.035	23.761 ± 2.014
C 16:1 palmitoleic acid	2.760 ± 0.014	2.340 ± 0.089
C 17:0 heptadecanoic acid	1.382 ± 0.032	0.524 ± 0.004
C 18:0 stearic acid	21.405 ± 0.912	17.855 ± 1.009
C 18:1 oleic acid	38.734 ± 3.012	41.572 ± 2.392
C 18:2 linoleic acid	4.276 ± 0.015	7.375 ± 0.012
C 18:3 linolenic acid	0.678 ± 0.001	0.702 ± 0.003
C 20:0 arachidic acid	0.319 ± 0.001	0.234 ± 0.002
C 20:1 gadoleic acid	0.289 ± 0.001	1.033 ± 0.004

N/D—not detected.

**Table 2:** Values of the activation energy of molecular movements in the oil and water phase of emulsions containing modified starches.

Starch Concentration (g/g)	Δ*E_a_* (kJ/mol)			
	Oil Phase		Water Phase	
	Beef Fat	Pork Fat	Beef Fat	Pork Fat
E 1420				
0.12	25.7 ± 0.6	23.5 ± 0.2	13.0 ± 0.3	12.3 ± 0.4
0.17	29.5 ± 0.4	27.3 ± 0.3	15.8 ± 0.5	19.5 ± 0.6
0.25	20.2 ± 0.3	17.4 ± 0.7	19.5 ± 0.2	13.7 ± 0.4
LU 1432				
0.12	21.1 ± 0.4	20.4 ± 0.7	28.9 ± 0.5	22.9 ± 0.2
0.17	27.7 ± 0.5	22.2 ± 0.3	31.2 ± 0.7	24.4 ± 0.4
0.25	22.1 ± 0.2	18.8 ± 0.5	10.9 ± 0.6	23.1 ± 0.5

## Data Availability

The datasets generated during and/or analyzed during the current study are available from the corresponding author on reasonable request.
